# Disparities in caesarean section prevalence and determinants across sub-Saharan Africa countries

**DOI:** 10.1186/s41256-018-0074-y

**Published:** 2018-07-02

**Authors:** Sanni Yaya, Olalekan A. Uthman, Agbessi Amouzou, Ghose Bishwajit

**Affiliations:** 1 School of International Development and Global Studies, University of Ottawa, Ottawa, ON K1N 6N5 Canada; 2 Warwick Centre for Applied Health Research and Delivery (WCAHRD), Division of Health Sciences, Warwick Medical School, University of Warwick, Coventry, CV4 7AL UK; 3 Bloomberg School of Public Health, Johns Hopkins University, Baltimore, MD 21205 USA

**Keywords:** Cesarean section, Delivery, Sub-Saharan Africa, Vaginal birth, Maternal health, Global health

## Abstract

**Background:**

Access to safe Cesarean section (C-section) in resource-constrained settings such as sub-Sahara Africa (SSA) region is a foremost approach to reduce maternal mortality. C-section is an obstetric operative procedure used appropriately to improve delivery outcomes. However, errors in the procedure have enormous potential harm that may outweigh the benefits. This study assessed the prevalence and determinants of C-section in several SSA countries. This study examined the prevalence and determinants associated with cesarean delivery in SSA countries.

**Methods:**

Secondary data of women of reproductive age (15-49 years) from the current Demographic and Health Survey (DHS) in 34 SSA countries was utilized in this study. The mode of delivery among women was the primary outcome variable. Percentage and descriptive statistics were used to conduct univariate analyses. Furthermore, multivariable multilevel logistic regression was used to investigate correlates of C-section among SSA women.

**Results:**

Results showed disparities in the percentage of C-section among women from 34 SSA countries. C-section at public healthcare settings ranged from 3% in Burkina Faso to 15.6% in Ghana. However, in private healthcare settings, C-section ranged from 0% in Sao Tome and Principe to 64.2% in Rwanda. Overall, C-section was 7.9% from public healthcare and 12.3% from private healthcare facilities respectively. In the adjusted regression model; women aged 35–49 had increase in the odds of C-section, while a unit increase in the number of children ever born had 17 and 20% significant reduction in the odds of C-section in public and private healthcare respectively. Assessing public healthcare settings; women from richer/richest households, male and large size children at birth had increase in the odds of C-section, while those from rich neighbourhood had reduction in the odds of C-section. In private healthcare settings, women with high decision making power and multiple births had increase in the odds of C-section, while those who attended ANC visits had significant reduction in the odds of C-section.

**Conclusion:**

The findings from this study would help formulate health policies and implement actions that would improve the outcome of C-section care. Monitoring of emergency obstetric care services is necessary to address issues connected to poor C-section outcomes in resource-constrained settings. Also training of medical personnel including midwives and nurses in emergency obstetric care, ensuring accessibility to life-saving drugs and supplies should be encouraged in health care system.

## Background

By the end of year 2015, the Sustainable Development Goals (SDGs) emerged with a target to bring a reduction in maternal mortality ratio (MMR) to less than 70 per 100,000 live births worldwide, and to ensure healthy lives for all at all ages by 2030 [1]. Despite the immense global interventions to reduce the problem of mother and child deaths due to complications in pregnancy and delivery, the magnitude of maternal mortality remains unabated specifically in sub-Sahara Africa region [2]. This implies the necessity to provide evidence-based, quality and high-impact maternal healthcare services, particularly; universal access to emergency gynecological and obstetric care be made a priority on the global health agenda. Predominantly, developing countries are known to account for approximately 99% of the estimated 303,000 maternal deaths that occur per year worldwide, where access to antenatal care, family planning, postnatal care and emergency obstetric services have been reported inadequate [3].

In the quest to achieve SDG-3, equity and equality in availability to emergency obstetric care including assisted vaginal delivery together with safe caesarean section (C-section) is exceedingly essential [4]. C-section is a known life-saving procedure for both mother and child. Contemporary obstetrics and gynecological practice for medical, economic and social reasons have observed higher rate of cesarean section universally [5, 6]. There is an increasing attention that C-section rates have consistently been on the rise, regardless of race, age, medical condition and gestational age. Global attention over such upsurge have persuaded World Health Organization (WHO) to recommend that C-section prevalence should not surpass 15% [7], with numerous evidence signifying that C-section prevalence beyond 15% were not linked to further reduction in maternal and child morbidity and mortality [8]. Notwithstanding, there are disparities in the prevalence of C-section in developing countries ranging between 2 and 39% [5, 9].

C-section has become a prominent indicator of accessing progress in emergency obstetric care, and a method to avert complications during labour and delivery [10]. The role of caesarean section in poor-resource settings is difficult to obviously describe. In many low- and middle-income countries, the tool has been reportedly underutilized especially among the disadvantaged populations, and overemployed by the privileged group, while no consideration has been adopted to ensure that universal access is obtained [11]. C-section has become a priority in agenda setting to curb the menace of maternal death through improved quality and use of services for the management and treatment of complications in pregnancy, labor and delivery [7]. Holistically, a foremost strategy in the reduction of maternal morbidity and mortality includes promoting skilled birth attendance or institutional delivery and utilization of prompt C-section as a remedy to salvage delivery crisis [12]. Evidence-based studies have shown numerous factors connected to consistent rise in the rate of C-section in many communities. As reported in a previous study, women with higher economic class underwent more C-section than women without formal education and those in low wealth index class. Furthermore, women who delivered in private health facilities are known to have more C-section than the women who utilized government owned health facilities [13].

Disparities exist in C-section trends across diverse populations, economic class amongst other factors in sub-Saharan Africa region and the world at large [14]. Elsewhere in sub-Sahara Africa, a study showed huge inequalities in C-section levels across diverse socio-environmental and demographic factors indicating differentials in accessibility to health care services [15]. More so, population based studies involving large number of women who delivered at referral centers in sub-Sahara Africa countries, identified individual woman factors related to C-section, while variations were noted across the rate of intrapartum, emergent and elective C-sections [16]. Considering the various types of C-section, prominent maternal risk factors are history of previous C-section, hypertension, premature rupture of membranes amongst others [16].

As a major abdominal surgery, C-section commonly happens under critical conditions as signified during fetal distress, hemorrhage, cephalopelvic disproportion and eclampsia. Unfortunately, this procedure is sometimes performed by incompetent and poorly trained personnel [11]. Despite it is being performed in overall high-risk women with baseline risk of adverse outcomes, C-section can worsen the outcomes through damage to pelvic organs, increased blood loss and increased risk of thromboembolism, to name but a few [11]. However, C-section can prevent stillbirth, maternal morbidity and complications-related mortality during pregnancy and childbirth. It is in the light of the above that this study examines the prevalence and correlates of caesarean section in sub-Sahara Africa region.

## Methods

### Data source

Secondary data analysis was conducted involving individual woman component of the dataset from the Demographic and Health Survey (DHS). Women from thirty-four (34) countries were included from SSA region where data had previously been collected 2008–2016. The selected countries were; Southern SSA countries (Lesotho, Namibia and Zimbabwe), Eastern SSA countries (Burundi, Comoros, Ethiopia, Kenya, Madagascar, Malawi, Mozambique, Rwanda, Tanzania, Uganda and Zambia), Western SSA countries (Benin, Burkina-Faso, Cameroon, Chad, Cote d’Ivoire, Gambia, Ghana, Guinea, Liberia, Mali, Niger, Nigeria, Sao Tome & Principe, Senegal, Sierra Leone and Togo) and Central SSA countries (Angola, Congo, Gabon and Democratic Republic of Congo), see the details of the sample size from Table 1. The data is publicly available and can be accessed from MEASURE DHS database at http://dhsprogram.com/data/available-datasets.cfm. DHS are usually implemented by the National Population Commission (NPC) with financial and technical assistance by ICF International provisioned through the USAID-funded MEASURE DHS program. DHS are nationally representative surveys that collect information on a wide range of topics such as demographic, socioeconomic, family planning and domestic violence amongst other areas. The survey covered men and women aged between 15 and 49 years, under-5 children residing in non-institutional settings and households. It involved multi-stage stratified cluster design based on a list of enumeration areas (EAs), which are systematically selected units from localities and constitute the Local Government Areas (LGAs). The LGAs are subdivisions of each of the administrative States (including the Federal Capital Territory) and classified under geographical zones/divisions [17].

**Table 1:** Description of Demographic and Health Surveys data by countries, in sub-Saharan Africa, 2007 to 2016

				HDI	
Country	Year	Number of births	Neighbourhood	Value	Category
Angola	2016	4087	537	0.533	High HDI
Benin	2012	7913	748	0.485	Moderate HDI
Burkina Faso	2010	7636	561	0.402	Low HDI
Burundi	2010	3197	375	0.404	Low HDI
Cameron	2011	5032	562	0.518	Moderate HDI
Chad	2014	2273	438	0.396	Low HDI
Comoros	2012	1575	252	0.727	High HDI
Congo	2011	5579	383	0.592	High HDI
Cote d’Ivoire	2012	3106	346	0.435	Low HDI
DR Congo	2013	8463	530	0.474	Moderate HDI
Ethiopia	2008	2699	548	0.448	Low HDI
Gabon	2012	3488	333	0.697	High HDI
Gambia	2013	3233	281	0.452	Low HDI
Ghana	2014	3091	422	0.579	High HDI
Guinea	2012	2029	276	0.414	Low HDI
Kenya	2014	8738	1504	0.555	High HDI
Lesotho	2014	1986	395	0.497	Moderate HDI
Liberia	2013	2987	319	0.427	Low HDI
Malawi	2016	12,478	850	0.476	Moderate HDI
Mali	2012	3947	390	0.442	Low HDI
Mozambique	2011	4962	591	0.418	Low HDI
Namibia	2013	3454	532	0.64	High HDI
Niger	2012	2990	404	0.353	Low HDI
Nigeria	2013	7591	818	0.527	High HDI
Rwanda	2014	5429	492	0.498	Moderate HDI
Senegal	2011	5367	390	0.494	Moderate HDI
Sierra Leone	2013	4966	429	0.42	Low HDI
Tanzania	2015	4634	600	0.531	High HDI
Togo	2014	3549	330	0.487	Moderate HDI
Uganda	2016	7747	696	0.493	Moderate HDI
Zambia	2013	6768	717	0.579	High HDI
Zimbabwe	2015	3973	399	0.516	Moderate HDI
Madagascar	2009	3241	546	0.512	Moderate HDI
Sao Tome & Principe	2008	1119	104	0.574	High HDI

### Variable measurement

#### Outcome variable

The dependent variable was the mode of delivery among women of reproductive age. Data about the method of last pregnancy delivery was collected in dichotomous form as either caesarean section or otherwise.

#### Independent variables

The explanatory factors include; current age of a respondent (15–19, 20–24, 25–29, 30–34, 35–39, 40–44, 45–49 years), place of residence (urban vs rural), respondent and partner’s educational attainment (none, primary, secondary and higher), religion (Christianity, Islam, traditional and other religion), sex of household head (male vs female), frequency of reading newspaper, frequency of listening to radio, frequency of watching television, birth type (singleton vs multiple), sex of child (male vs female), total children ever born, age of respondents at first birth, number of antenatal visits, size of child at birth (very large, larger than average, average, smaller than average, very small) and employment status (employed vs unemployed), women’s decision making power; was evaluated with questions on who made decisions about women’s own health care, household purchases, visits to family members and husband earnings. All decision components were categorized into a decision made by husband or other person, a decision made together with the woman, or a decision made by the woman only. In addition, on wealth index; the calculation of household socioeconomic level involved the use of items such as possession of TV, radio, bicycle, type of floor, roof, toilet facility, water source and so forth. The principal component analysis (PCA) was used in assigning a score which were then summed and standardized for the households. The standardized scores places the households on a continuous scale based on relative wealth scores. The scores were thus categorized into quintiles to rank the household as poorest/poorer/middle/richer/richest. The place of delivery was grouped as private vs public health facility.

Neighbourhood-level factor was operationalized with a principal component analysis using the proportion of respondents with: no formal education, rural resident, unemployed and living below the poverty level (asset index below 20% poorest quintile). A standardized score with mean 0 and standard deviation 1 was computed from this index; with higher scores indicative of lower socio-economic position (SEP). We grouped the scores into tertiles to allow for nonlinear effects and provide results that were more readily interpretable in the policy arena.

Country-level factor included human development index, a measure of country’s intensity of deprivation, which is the average percentage of deprivation experienced by people in multidimensional poverty. The country-level variables were also grouped into tertiles (low, middle and high levels).

### Ethical approval

We conducted the analyses using publicly available data from demographic health surveys. Prior to each interview, participants gave informed consent to participate in the survey. DHS Program is consistent with the standards for ensuring the protection of respondents’ privacy. ICF International ensures that the survey complies with the U.S. Department of Health and Human Services regulations for the respect of human subjects. No further approval was required for this study since the data is secondary and is available in the public domain. More details about data and ethical standards are available at: http://goo.gl/ny8T6X.

### Data management plan

The baseline socio-economic, demographic and other characteristics of respondents were computed using summary statistics together with percentages. The complex survey module (svyset) was used to account for sample weight. In addition, the association between explanatory variables and C-section among women was investigated using multivariable multilevel fixed-and-random effect logistic regression was used to obtain stratified models for public and private health facilities. The results of fixed effects (measures of association) were reported as odds ratios (ORs) with their 95% credible intervals (CrIs). Significance level was set at 5%. Data analyses was done using STATA version 14.0 (Statacorp, College Station, Texas, United States of America).

## Results

### Sample characteristics

**Fig. 1:**
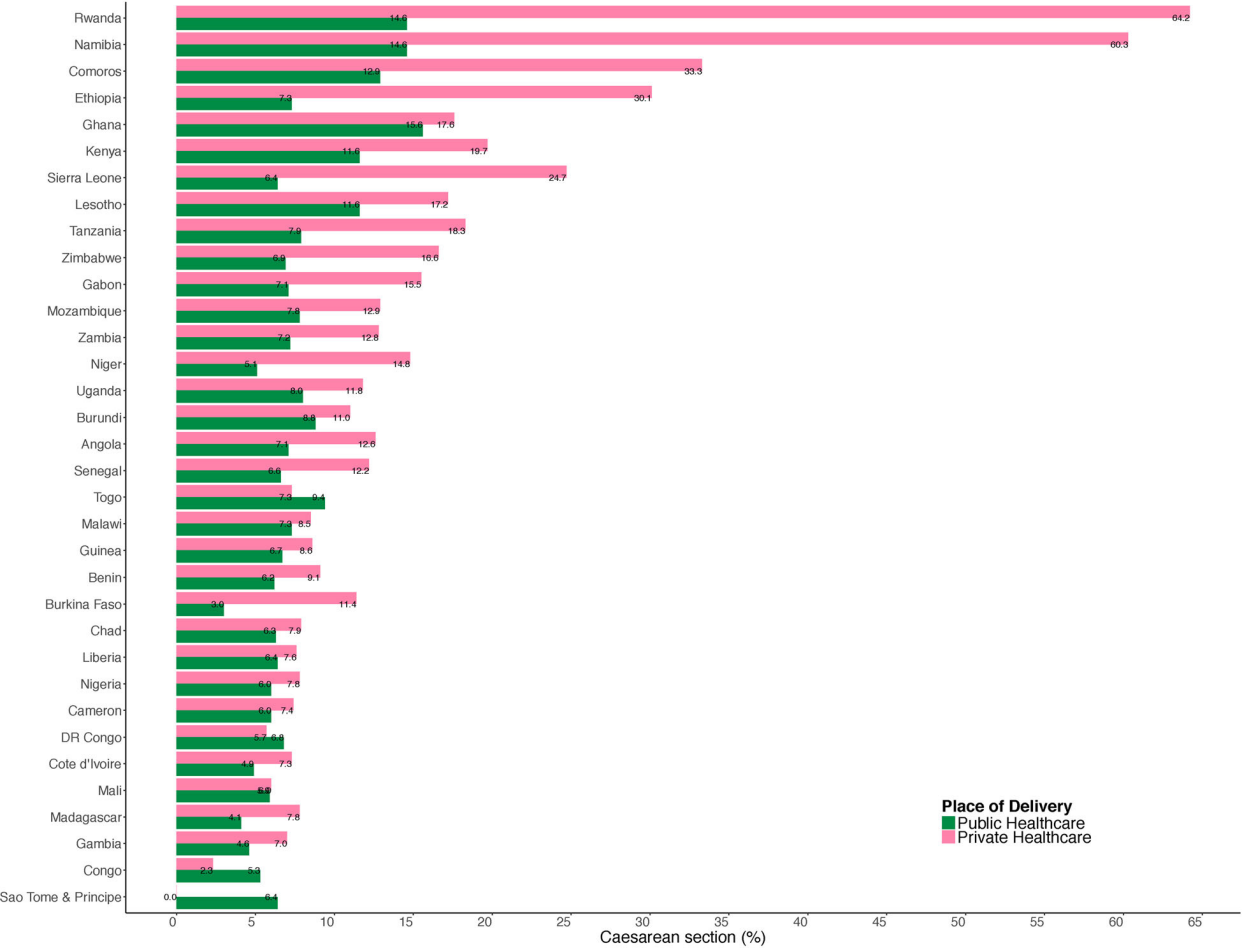
Caesarean section among women in sub-Saharan countries

We analysed information on 159,327 respondents from 32 countries in sub-Saharan Africa (Table 1). Table 1 shows the countries, year of data collection, and the surveys characteristics. The median number of neighbourhoods sampled was 434, ranging from 104 in Sao Tome & Principe to 1504 in Kenya. The median number of respondents was 3960 (range: 1119 to 12,478). As shown in Fig. 1, there was a wide variation in the percentage of C-Section. The characteristics of the pooled sample is shown in Table 2.

**Table 2:** Summary of pooled sample characteristics of the Demographic and Health Surveys data in sub-Saharan Africa

Caesarean section			
	Overall (%)	Public healthcare (%)	Private healthcare (%)
	159,327	139,882	19,445
CS		7.9	12.3
Age (%)			
15–24	31.5	7.1	9.1
25–34	46.4	8.0	12.8
35–49	22.1	8.7	15.3
Wealth (%)			
Poorest	14.5	5.1	6.9
Poorer	17.5	5.8	7.5
Middle	19.7	6.6	8.2
Richer	23.0	7.9	9.8
Richest	25.3	12.5	17.6
Maternal’s education (%)			
No education	27.5	5.3	7.7
Primary	37.0	7.3	9.2
Secondary+	35.5	10.8	15.4
Paternal’s education (%)			
No education	25.8	5.1	6.7
Primary	30.1	6.9	8.2
Secondary+	44.1	9.6	13.7
Religion			
Christianity	70.7	8.5	12.9
Islam	23.9	6.6	9.0
Others	5.4	6.4	7.7
Media access			
0	23.3	5.7	6.4
1	31.1	6.6	8.8
2	30.2	8.7	11.7
3	15.3	12.7	20.7
Household head			
Female	77.0	9.2	12.1
Male	23.0	7.5	12.9
Currently working			
Yes	37.9	7.8	12.7
No	62.1	7.9	12.1
Decision making power			
Low	30.7	6.6	8.9
Medium	36.9	8.8	12.3
High	32.4	9.7	15.9
Sex of child			
Male	48.9	8.4	11.9
Female	51.1	7.3	12.7
Multiple birth			
No	99.9	7.9	12.3
Yes	0.1	12.5	25.6
Large size at birth			
Yes	60.6	7.1	11.9
No	39.4	8.6	11.1
Antenatal care			
None	4.1	6.4	10.6
1 to 4 visits	49.5	7.5	10.2
5 to 8 visits	33.7	8.8	13.7
8 or more visits	12.7	11.5	16.0
Neighbourhood SES (%)			
Tertile 1 (least disadvantaged)	38.8	10.9	14.6
Tertile 2	36.3	7.3	9.9
Tertile 3 (most disadvantaged)	24.9	4.6	7.1
Human Development Index (%)			
Low HDI	27.1	5.5	9.7
Moderate HDI	41.2	8.1	10.1
High HDI	31.7	9.7	15.6

### C-section at public healthcare settings

The prevalence of c-section ranged from 3% in Burkina Faso to 15.6% in Ghana (Fig. 1). The results of multilevel model is shown in Table 3**.** In the fully adjusted model controlling for the effects of individual, neighbourhood and country level factors, maternal age, wealth, sex of child, number of children and size of the baby at birth were significantly associated with odds of c-section. Women aged 35 to 49 years old were more likely to have had c-Section to those aged 15 to 24 years old (OR = 2.75, 95% CrI 2.33to 3.22). Women from the richest households were almost as twice as likely to have had c-Section than those from poorest households (OR = 1.97, 95% CrI 1.54 to 2.55). Male children were more likely to have been delivered via c-section than female children (OR = 1.21, 95% CrI 1.10 to 1.33. The odds of c-section decreased with increasing number of children ever born (OR = 0.83, 95% CrI 0.81 to 0.86). Children large size at birth were more likely to have been delivered via c-section (OR = 1.15, 95% CrI 1.05 to 1.26).

**Table 3:** Individual compositional and contextual factors associated with Caesarean section in sub-Saharan Africa identified by multivariable multilevel logistic regression models, Demographic and Health Surveys data

	Place of Delivery	
	Public healthcare (OR, 95% CrI)	Private healthcare (OR, 95% CrI)
Age (%)		
15–24	1 (reference)	1 (reference)
25–34	1.50 (1.31 to 1.72)	2.32 (1.58 to 3.47)
35–49	2.75 (2.33 to 3.22)	4.31 (2.83 to 6.67)
Wealth (%)		
Poorest	1 (reference)	1 (reference)
Poorer	1.07 (0.87 to 1.33)	1.75 (0.78 to 3.69)
Middle	1.14 (0.91 to 1.44)	1.24 (0.53 to 2.68)
Richer	1.34 (1.09 to 1.70)	1.48 (0.65 to 3.21)
Richest	1.97 (1.54 to 2.55)	2.08 (0.89 to 4.19)
Maternal’s education (%)		
No education	1 (reference)	1 (reference)
Primary	1.03 (0.89 to 1.18)	1.15 (0.71 to 1.79)
Secondary+	1.09 (0.92 to 1.27)	1.61 (0.96 to 2.43)
Paternal’s education (%)		
No education	1 (reference)	1 (reference)
Primary	1.04 (0.89 to 1.22)	0.86 (0.47 to 1.46)
Secondary+	1.13 (0.95 to 1.32)	1.22 (0.68 to 2.01)
Religion		
Christianity	1 (reference)	1 (reference)
Islam	1.08 (0.94 to 1.25)	0.54 (0.37 to 0.75)
Others	1.00 (0.78 to 1.23)	0.62 (0.30 to 1.08)
Media access	1.02 (0.96 to 1.09)	1.08 (0.95 to 1.23)
Female Household head	1.01 (0.89 to 1.13)	0.94 (0.71 to 1.19)
Currently working	0.92 (0.76 to 1.07)	1.02 (0.63 to 1.75)
Decision making power		
Low	1 (reference)	1 (reference)
Medium	1.05 (0.93 to 1.18)	1.20 (0.91 to 1.57)
High	1.06 (0.93 to 1.20)	1.52 (1.14 to 1.97)
Sex of child (male vs female)	1.21 (1.10 to 1.33)	1.03 (0.85 to 1.23)
Multiple birth	1.01 (0.09 to 3.24)	22.94 (4.24 to 77.55)
Number of children	0.83 (0.81 to 0.86)	0.80 (0.75 to 0.86)
Large size at birth	1.15 (1.05 to 1.26)	1.05 (0.86 to 1.27)
Antenatal care		
None	1 (reference)	1 (reference)
1 to 4 visits	0.86 (0.60 to 1.08)	0.51 (0.30 to 0.94)
5 to 8 visits	0.98 (0.68 to 1.24)	0.60 (0.35 to 1.12)
8 or more visits	1.28 (0.90 to 1.66)	0.84 (0.48 to 1.65)
Neighbourhood SES (%)		
Tertile 1 (least disadvantaged)	1 (reference)	1 (reference)
Tertile 2	0.86 (0.77 to 0.98)	1.00 (0.76 to 1.28)
Tertile 3 (most disadvantaged)	0.79 (0.67 to 0.64)	1.04 (0.55 to 1.74)
Human Development Index (%)		
Low HDI	1 (reference)	1 (reference)
Moderate HDI	1.26 (0.91 to 1.69)	0.94 (0.36 to 1.75)
High HDI	1.26 (0.84 to 1.70)	1.09 (0.48 to 2.05)
Random-effect		
*Country-level*		
Variance (95% CrI)	0.18 (0.10 to 0.31)	0.46 (0.21 to 0.89)
*Neighbourhood-level*		
Variance (95% CrI)	0.00 (0.00 to 0.00)	0.00 (0.00 to 0.00)

*OR* Odds ratio, *CrI* Credible interval, *MOR* Median odds ratio, *VPC* Variance partition coefficient, *DIC* Bayesian Deviance Information Criteria

### C-section at private healthcare settings

The results of multilevel model is shown in Table 3**.** In the fully adjusted model controlling for the effects of individual, neighbourhood and country level factors, maternal age, decision making power, multiple birth and number of children were significantly associated with odds of c-section. Women aged 35 to 49 years old were more likely to have had c-Section to those aged 15 to 24 years old (OR = 4.31, 95% CrI 2.83 to 6.67). Women with high decision making power were more likely to have had c-section (OR = 1.52, 95% CrI 1.14 to 1.97). The odds of c-section decreased with increasing number of children ever born (OR = 0.80, 95% CrI 0.75 to 0.86).

### Percentage of caesarean section among women in sub-Sahara Africa countries

Figure 1 presents the percentage of C-section among women from 34 SSA countries. The highest percentage (64.2%) of C-section was reported among deliveries in Rwanda, while Namibia reported about 60.3%, Comoros and Ethiopia reported 33.3 and 30.1% respectively from private settings. Overall, public healthcare settings reported lower C-section in several SSA countries including Sao Tome & Principe (0%), Congo (2.3%), Burkina-Faso (3%) amongst others (see fig. 1 for details).

## Discussion

The focus of this study was to investigate the prevalence and determinants of C-section mode of delivery in SSA countries using current nationally representative data from DHS conducted between 2008 and 2016. The findings from our analysis revealed disparities in the prevalence of C-section across various countries, while some reported substantial increase during the study period, some countries had very low percentage of C-section. The prevalences obtained were similar to previous reports from developing countries [18–20]. According to WHO, the prevalence of C-section in any population should be within the interval of 5–15%, however, some countries in SSA were found to have below the minimum 5% recommended by public and private healthcare facilities. Studies have shown that the prominent reasons for the low coverage of C-sections were insufficient provision of equipment and medicines in the available emergency obstetric health units, lack of skilled birth attendants, unavailability of life-saving obstetrics services, long distances and poor landscapes without proper transportation could cause major geographic barrier in access to emergency obstetric care [21, 22]. Conversely, despite the reasons for the high prevalence in some countries are not completely known due to multifactorial nature, it could be connected to supply-side and demand-side factors. Maternal factors, such as education and other characteristics driven by health professionals, health care system, awareness, perception and socio-economic factors could be responsible.

The multilevel logistic regression model used to examine significant predictors of C-section, indicated that age, wealth index, parity (number of children ever born), sex of child, size of child at birth, neighbourhood socioeconomic status, women’s decision making power, multiple births (i.e: twins, triplets etc.) and antenatal care visits were significantly associated with having a C-section model of delivery. Consistent with previous studies [23, 24], our findings showed that women who belong to the poorest households had lower odds of C-section than those from rich households in SSA countries. The current data have confirmed poverty as a major factor responsible for the low utilization of C-section among women. The cost implication of assessing a crucial life-saving procedure such as C-section is a major factor that could hinder the process of achieving equality health care services utilization as evident in this study. However, women from least disadvantaged neighbourhood had significant reduction in the odds of C-section. Affordability of adequate antenatal care visits; a medium of behavioural change communication through feeding habits, exercising and warning signs at onset of pregnancy-related complications enlightenment could help reduce the occurrence on emergency C-section. In this study, older women had higher C-section, which could be due to complications resulting from advanced reproductive age or due to life time C-section data used for this study. This is consistent with the findings from previous study [25].

Further, women with large number of children had less of C-section; the experience from several child births could reduce the risk of pregnancy related and intrapartum complications among the multiparous women. Child factors associated with C-section were sex, size of baby at birth and type of birth. Multiple births such as twins, triplets or more and large baby size or weight could cause cephalo pelvic disproportion or malposition. Child sex was also significantly associated with C-section among women in SSA countries. Previous studies have reported similar factors significantly associated with C-section [6, 26].

### Strength and limitation

This study used large sample size involving multiple nationally representative datasets from several countries in SSA to investigate prevalence and correlates of C-section in sub-Sahara Africa region. Nonetheless, the data lacks information relating to clinical indications for C-sections; as the data did not distinguish between elective and emergency C-sections. Also, the use of this information for decision-making and comparison should consider the cross-sectional nature of the data which is inadequate to sufficiently establish causality. Maternal empowerment has been known to affect several health care services. In this study, women’s decision making power was significantly associated with increased C-section. In an effort to educate women about their birthing rights and options, guides are provided for women on healthy birth mode [27]. Educating expectant mothers with information on pregnancy, delivery and hospital C-section rates could assist women in making informed choices, leading to increase in C-section.

## Conclusion

In practice, healthcare system is grouped into public and private hospitals with specific features according to the country. The differences in C-section prevalence between public and private healthcare settings are either due to the difference in prenatal and delivery care between these two settings that could influence the delivery outcome or the preference of patient mode of delivery. The high prevalence of C-section in private healthcare settings is a broad concept connected with several factors. Therefore, the interventions and programs should be targeted to address both maternal preference and professional attitude towards the mode of delivery. Educational interventions to improve quality of painless labour and vaginal delivery should be introduced in both public and private healthcare settings to lower potential C-section (elective) rate. Women’s awareness towards social beliefs as C-section (elective) is safer than normal (vaginal) delivery and information regarding complications of C-section and their outcomes should be enhanced. Active involvement of the policy sector is needed to strengthen equity and universal health coverage in maternal healthcare. The results suggest the need for accurate and timely screening of women during obstetric care and, the choice of performing C-section should be based on clear and well-supported justifications.

## Abbreviations

ANC: Antenatal care
C-Section: Cesarean Section
DHS: Demographic Health Survey
IRB: Institutional Review Board
MMR: Maternal mortality ratio
SDGs: Sustainable Development Goals
SSA: Sub-Saharan Africa
WHO: World Health Organization

## References

[1] UN General Assembly, Transforming our world: the 2030 Agenda for Sustainable Development, 21 October 2015, A/RES/70/1. Available at: http://www.refworld.org/docid/57b6e3e44.html. Accessed 3 Mar 2018.

[2] Yaya S, Bishwajit G, Shah V (2016). Wealth, education and urban–rural inequality and maternal healthcare service usage in Malawi. BMJ Global Health.

[3] WHO (2010). Trends in Maternal Mortality: 1990 to 2008.

[4] Chu K, Cortier H, Maldonado F, Mashant T, Ford N (2012). Cesarean section rates and indications in sub-Saharan Africa: a multi-country study from Medecins sans Frontieres. PLoS One.

[5] Lauer JA, Betrán AP, Merialdi M, Wojdyla D (2007). Rates of caesarean section: analysis of global, regional and national estimates. Paediatr Perinatal Epidemiol.

[6] Abebe FE, Gebeyehu AW, Kidane AN, Eyassu GA (2016). Factors leading to cesarean section delivery at Felegehiwot referral hospital, Northwest Ethiopia: a retrospective record review. Reprod Health.

[7] WHO. Monitoring Emergency Obstetric Care: A Handbook. Geneva, Switzerland; 2009.

[8] Adnan A, Abu O, Suleiman H, Abu A (2012). Frequency Rate and Indications of Cesarean Sections at Prince Zaid Bin Al Hussein Hospital – Jordan. J Med SciClin Res..

[9] Shamshad B (2008). Factors leading to increased cesarean section rate. Gomal J Med Sci.

[10] Miller S, Abalos E, Chamillard M, Ciapponi A, Colaci D, Comande AD (2016). Beyond too little, too late and too much, too soon: a pathway towards evidence-based, respectful maternity care worldwide. Lancet.

[11] Fawcus S, Pattinson RC, Moodley J, Moran NF, Schoon MG, Mhanga RE, Baloyi S, Bekker E, Gebhardt GS (2016). Maternal deaths from bleeding associated with caesarean delivery: a national emergency. SAMJ.

[12] Stanton C, Blanc A, Croft T, Choi Y (2007). Skilled care at birth in the developing world: progress to date and strategies for expanding coverage. J Biosoc Sci.

[13] Gebremedhin S (2014). Trend and socio-demographic differentials of cesarean section rate in Addis Ababa, Ethiopia: analysis based on Ethiopia demographic and health surveys data. Reprod Health.

[14] Cavallaro FL, Cresswell JA, Franca GVA, Victora CG, Barros AJD, Ronsmans C (2013). Trends in cesarean delivery by country and wealth quintile: cross-sectional surveys in southern Asia and sub-Saharan Africa. Bull World Health Organ.

[15] Nilsen C, Ostbye T, Daltveit AK, Mmbaga BT, Sandoy IF (2014). Trends in sociodemographicfactors associated with caesarean section at a Tanzanian referral hospital, 2000 to 2013. Int J Equity Health.

[16] Briand V, Dumont A, Abrahamowicz M, Traore M, Watier L, Fournier P (2012). Individual and institutional determinants of cesarean section in referral hospitals in Senegal and Mali: a cross-sectional epidemiological survey. BMC Pregnancy Childbirth.

[17] Rutstein SO, Rojas G. Guide to DHS Statistics Demographic and Health Survey’s DHS Toolkit of methodology for the MEASURE DHS Phase III project, implemented from 2008–2013: United States Agency for International Development (USAID). MEASURE DHS/ICF International.

[18] Mumtaz S, Bahk J, Khang YH (2017). Rising trends and inequalities in cesarean section rates in Pakistan: evidence from Pakistan demographic and health surveys, 1990-2013. PLoS One.

[19] Betran AP, Ye J, Moller AB, Zhang J, Gumezoglu AM, Torloni MR (2016). The increasing trend in cesarean section rates: global, regional, and national estimates: 1990-2014. PLoS One.

[20] Neuman M, Alcock G, Azad K, Kuddus A, Osrin D, More NS (2014). Prevalence and determinants of caesarean section in private and public health facilities in underserved south Asian communities: cross-sectional analysis of data from Bangladesh, India and Nepal. BMJ Open.

[21] Bailey P, Paxton A, Lobis S, Fry D (2006). The availability of life-saving obstetric services in developing countries: an in-depth look at the signal functions for emergency obstetric care. Int J Gynaecol Obstet.

[22] Gabrysch S, Cousens S, Cox J, Campbell OM (2011). The influence of distance and level of care on delivery place in rural Zambia: a study of linked national data in a geographic information system. PLoS Med.

[23] Anwar I, Nababan HY, Mostari S, Rahman A, Khan JA (2015). Trends and inequities in use of maternal health care services in Bangladesh, 1991-2011. PLoS One.

[24] Ronsmans C, Holtz S, Stanton C (2006). Socioeconomic differentials in caesarean rates in developing countries: a retrospective analysis. Lancet.

[25] Khan MN, Islam MM, Shariff AA, Alam MM, Rahman MM (2017). Socio-demographic predictors and average annual rates of caesarean section in Bangladesh between 2004 and 2014. PLoS One.

[26] Vieira GO, Fernandes LG, de Oliveira NF, Silva LR, Vieira TDO. Factors associated with cesarean delivery in public and private hospitals in a city of northeastern Brazil: a cross-sectional study. BMC Pregnancy Childbirth. 2015;15:132. 10.1186/s12884-015-0570-8 PMC4457097 26043857

[27] McAllister E (2008). Transparency in maternity care: empowering women to make educated choices. J Perinat Educ.

